# Millennium Cohort Study: cohort profile and data resource update

**DOI:** 10.1136/bmjmed-2025-002429

**Published:** 2026-09-18

**Authors:** Aase Villadsen, Emla Fitzsimons

**Affiliations:** 1 UCL Centre for Longitudinal Studies, University College London, London WC1H 0AL, UK

**Keywords:** Public health, Epidemiology

## Abstract

This cohort profile gives a comprehensive overview of the Millennium Cohort Study (MCS), a nationally representative study of children born in the UK during 2000-02. The study is for researchers in public health and other disciplines who seek a comprehensive overview of the MCS and its data resources. This profile describes the study design, sampling strategy, population characteristics, breadth of measures collected, the availability of biological samples and linked administrative data, approaches to missing data and attrition, and key resources for navigating and accessing the datasets. Earlier cohort profiles are also updated here, and the full range of data resources currently available (through to the most recent survey of participant at age 23 years) is documented.

**Key messages**

- This profile extends coverage to the most recent survey of participants aged 23 years in a large, nationally representative UK birth cohort of more than 19 000 individuals born during 2000-02.
- The cohort study is multidisciplinary in scope, encompassing social, economic, familial, biological, cognitive, and health domains.
- This study incorporates data from cohort members, parents, siblings, teachers, and partners, along with biological samples and linked administrative data.
- As one of the most widely used data sources on children and families in the UK, by academics, central government, think tanks, and the non-profit sector, MCS findings have informed numerous policy areas.
- With data now extending into early adulthood, MCS provides a platform for understanding development over the lifespan and could inform public health, social policy, and practice in multiple areas.

## Introduction

**Figure 1:**
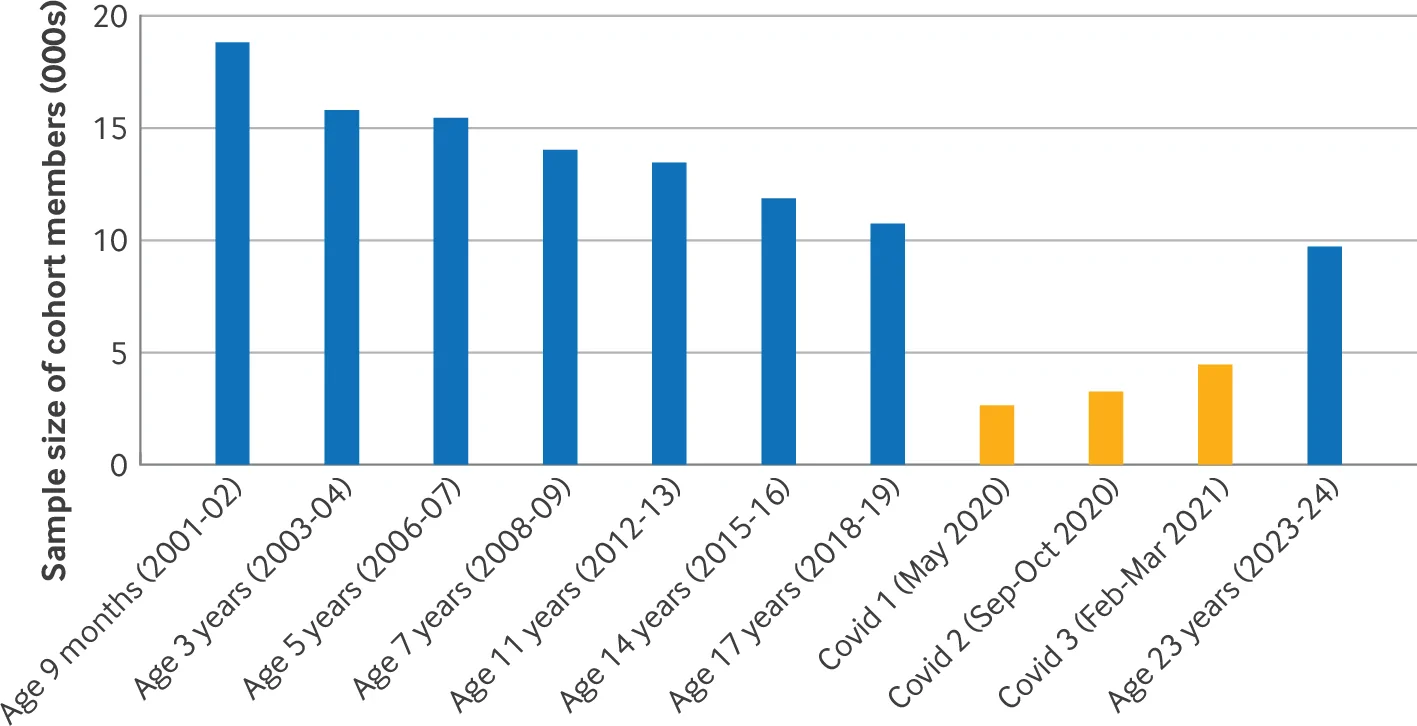
Summary of sample size of each survey from participant age 9 months to 23 years. Yellow bars show shorter surveys conducted during the covid-19 pandemic

 The Millennium Cohort Study (MCS) is an ongoing longitudinal study tracking more than 19 000 individuals born in the UK between September 2000 and January 2002. The study follows the style of earlier national birth cohorts from 1946, 1958, and 1970, and fills the generational gap by focusing on a more recent cohort. Unlike its predecessors—which began with a primary focus on child health—the MCS was designed as a multidisciplinary study to capture the broader social landscape of human development, with a strong emphasis on data capture in the early years. The first survey was conducted when cohort members were 9 months old, followed by sweeps at ages 3, 5, 7, 11, 14, 17, and 23 years. Three additional shorter surveys were done during the covid-19 pandemic. Figure 1 summarises all surveys to date and the sample sizes achieved. The vast majority of data from the study are openly available to bona fide researchers through the UK Data Service. This cohort profile updates the three previous profiles, which described the MCS up to ages 11, 14, and 17 years, respectively,^1–3^ and provides the first comprehensive account of the cohort and all data resources through to the most recent sweep at age 23 years.

## Population

The MCS was designed to represent children born in the UK at the start of the 21st century. The eligible population included all children born between 1 September 2000 and 31 August 2001 in England and Wales, and between 24 November 2000 and 11 January 2002 in Scotland and Northern Ireland, who were alive, residing in the UK, and had parents who were eligible for child benefit at 9 months of age.^4^ The sampling frame was the child benefit register, a scheme that allows anyone in the UK with a child to register to receive non-means tested monthly payments to help with the cost of bringing up a child. This register had near universal coverage (an estimated 97% of families with children) at the time of sampling, with little variation by family income.^5^ Geographical areas (electoral wards as they stood before updating at the 2001 census) were selected, and all eligible children within them comprised the potential recruitment group. To increase analytical power for typically underrepresented groups, some of which are known to be less likely to respond to invitations to participate and more likely to later leave the study, the design oversampled children in socioeconomically disadvantaged areas (using the child poverty index), children from areas with a large ethnic minority population (in England) using the 1991 population census, and children living in Scotland, Wales, and Northern Ireland. As a result, survey weights are needed to obtain accurate population estimates. The sample of wards was selected using probability (or random) methods of selection, combined with stratification and clustering. Division of the estimated achieved sample across strata was half in advantaged wards and half in disadvantaged wards in Wales, Scotland, and Northern Ireland, and half in advantaged wards and a quarter each in the wards with high levels of ethnic group diversity and socioeconomic disadvantage, respectively, in England. A detailed technical report on the sampling strategy is available.^4^

The first survey of participtant at age 9 months achieved a sample of 18 552 families, including 246 pairs of twins and 10 sets of triplets (18 818 children). A further 692 families (701 children) were added at age 3 years; these families had been eligible initially but were not contacted at 9 months because of recent address changes not yet reflected in the child benefit register. In total, 19 243 families (19 517 children) took part in the first or second sweep, representing a 75% response rate from 25 657 eligible families.^3^

**Figure 2:**
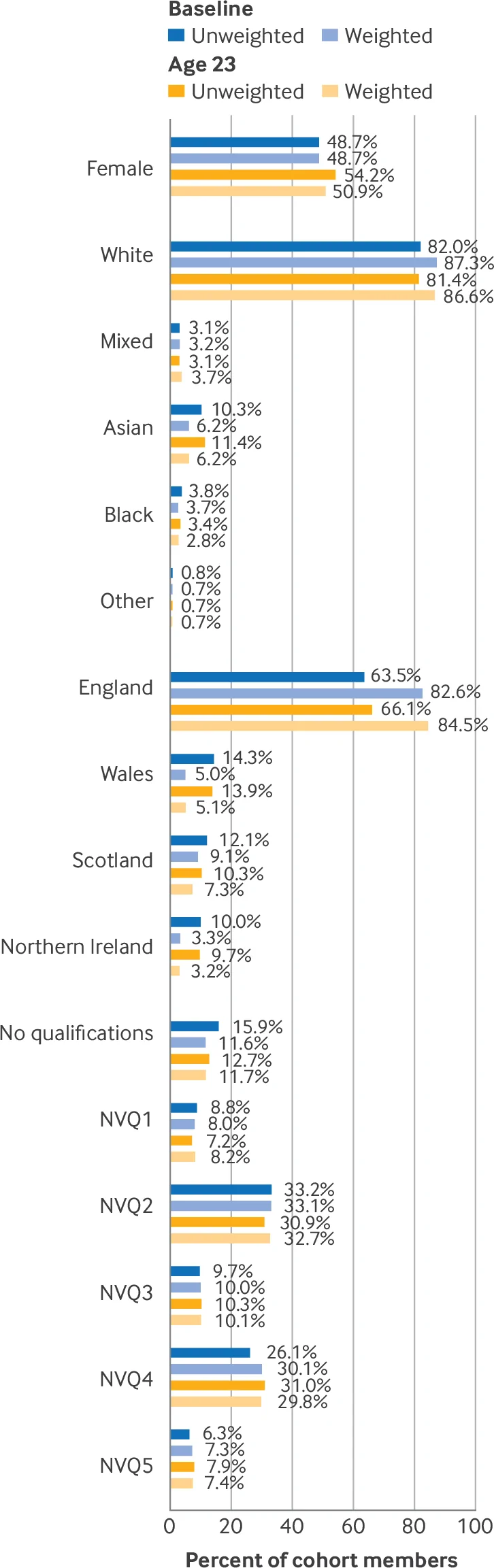
Sample characteristics at baseline and at participant age 23 years. NVQ=National Vocational Qualification

Figure 2 (pale blue bars) shows weighted baseline characteristics at cohort member age 9 months (or age 3 years for late entrants). Girls accounted for 48.7% of the sample. Around 87% of cohort members were white, 6.2% Asian, 2.7% black, 3.2% of mixed ethnicity, and 0.7% of other ethnicity. Geographically, 82.6% lived in England, 9.1% in Scotland, 5.0% in Wales, and 3.3% in Northern Ireland. Regarding household education, 11.6% had parents with no qualifications, whereas about 37% had a university degree or higher (NVQ 4/5). Unweighted data (dark blue bars) demonstrate the intended oversampling of disadvantaged families, ethnic minority groups, and the smaller UK nations.

Comparisons can be made to the 2001 census (England and Wales only) to demonstrate how the MCS (weighted baseline data) compares to the national population on demographic characteristics. For ethnicity (calculated from dataset ST101: sex and age by ethnic group) the MCS is consistent with population statistics for 0-4 year olds (85.7% white, 3.8% of mixed race, 6.7% Asian, 3.1% black, and 0.8% of other ethnicity). As for educational attainment, based on census dataset ST105 (sex and age by highest level of qualification gained), the proportion of households with an undergraduate degree or higher in the MCS is somewhat higher than the 25% observed among all 30-34 year olds in the census (the approximate average age of parents in the MCS).

Against other benchmarks, the MCS shows similar levels of children being overweight or obese in early childhood compared with national levels. In the 2006/2007 National Child Measurement Programme, 435 927 (22.9%) reception age children (aged 4 to 5 years) in England were overweight or obese,^6^ compared with 20.4% of English children in the MCS at age 5 years.

## Measures

The MCS is a multidisciplinary, multi-informant longitudinal study that follows participants from infancy into early adulthood. Across eight survey sweeps, the study generates a wealth of data spanning social, economic, familial, biological, cognitive, and health domains from the ages of 9 months to 23 years. Table 1 summarises the breadth of topic areas covered and the modes of data collection used across sweeps. Table 2 details the survey modes used.

**Table 1:** Information collected in the MCS surveys

Survey information collected	Cohort member age at survey							
	**9 months**	3 years	5 years	7 years	11 years	14 years	17 years	23 years
**Main parent and partner questionnaires**								
Information on parents and family context:								
Household members and composition								
Ethnicity*, religion, languages spoken at home	X	X	X	X	X	X		
Housing	X	X	X	X	X	X	X	
Local area	X	X	X	X	X	X		
Employment and income	X	X	X	X	X	X	X	
Education	X	X	X	X	X	X		
Parenting activities	X	X	X	X	X	X	X	
Family relationships	X	X	X	X	X	X		
Mental health	X	X	X	X	X	X	X	
Physical health	X	X	X	X	X	X	X	
Information on cohort members:								
Demographics (date of birth, sex, ethnicity)*	X	X	X	X	X			
Pregnancy, labour and delivery, baby's development	X							
Physical health	X	X	X	X	X	X		
Mental health and behaviour		X	X	X	X	X	X	
Childcare	X	X	X	X	X			
Schooling/Education			X	X	X	X	X	
Hobbies/leisure activities			X	X	X			
**Cohort member questionnaires**								
Hobbies/leisure activities				X	X	X	X	
Friendships				X	X	X	X	
School				X	X	X	X	
Mental health and well-being				X	X	X	X	X
Future aspirations				X	X	X	X	X
Family relationships					X	X	X	X
Local area					X	X	X	
Bullying and victimisation					X	X	X	X
Substance use and addictive behaviours					X	X	X	X
Crime and antisocial behaviours					X	X	X	X
Social media					X	X	X	X
Attitudes						X	X	X
Non-resident parents						X	X	
Physical health						X	X	X
Puberty and sexual relationships						X	X	X
Body image						X	X	X
Self-harm						X	X	X
Pregnancy						X	X	X
Sexual and gender identity						X	X	X
Ethnicity						X		
Employment							X	X
Income							X	X
Education and training after the age of 16								X
Household members and composition								X
Marital status								X
Children								X
Housing								X
Contraception								X
Partner relationship								
**Measurements**								
Physical measurements		X	X	X	X	X	X	X^†^
Cognitive assessments		X	X	X	X	X	X	X
Activity monitor (accelerometer)				X		X		
Time use diary						X		
**CM's cohabiting partner**								
Country and ethnicity								X
Education								X
Employment								X
Income								X
Physical health								X
Mental health								X
Substance use and addictive behaviours								X
Parents and family structure								X
Parental education and occupation								X
**Older sibling (age 10-15 years) ** ^‡^								
Extracurricular activities, life at home, health, school, bullying and victimisation, and anti-social behaviours (England only)		X	X					
**Teachers**								
Child behaviour and development			X^§^	X	X^¶^			
Educational environment				X	X^¶^			
**Interviewer**								
Home observations		X	X					
Neighbourhood observations		X	X					
**Biological samples**								
Saliva for virus antibodies		X						
Saliva for DNA extraction (CM and parents)						X		
Milk teeth (posted by parents) to assess lead exposure				X				

* Asked if not known from previous sweeps, or to check and verify.

† Online mode mainly, so weight, height, and body fat taken from a small sample only.

‡ Maximum of two older siblings selected.

§ Teachers in Wales, Scotland, and Northern Ireland completed Foundation Stage Profiles on children’s development and learning. In England this information was obtained using linked data (table 3).

¶ Teachers in England and Wales only.

**Table 2:** Survey mode used for participants at each survey

Survery mode by respondent	Survey mode used by participant age at survey sweep							
	9 months	3 years	5 years	7 years	11 years	14 years	17 years	23 years
Main parent questionnaires	Face to face, CAPI, CASI (80 mins)	Face to face, CAPI, CASI (63 mins)	Face to face, CAPI, CASI	Face to face, CAPI, CASI (61 mins)	Face to face, CAPI, CASI (62 mins)	Face to face, CAPI, CASI (36 mins)	Face to face, and online CAWI and paper* (20 mins) and CAPI†	^‡^
Resident partner questionnaires	CAPI, CASI (35 mins)	CAPI, CASI (22 Mins)	CAPI, CASI	CAPI, CASI (21 Mins)	CAPI, CASI (24 Mins)	CAPI, CASI (20 Mins)	CAWI (15 mins)	
Cohort member questionnaires				Face to face, paper (15 mins)	Face to face, paper (30 mins)	Face to face, CASI (45 mins)	Face to face and online, CAPI+CASI + CAWI (45 min)	Online or face to face, (60/75 mins) CAPI+CASI
Cohort member's cohabiting partner								Online CAWI
Older sibling (age 10-15 years) ^**§**^		Paper	Paper					
Teachers			Postal	Postal	Postal and phone (15 mins)			

Length of survey reported where available.

* SDQ questionnaire could also be completed by the partner parent.

† CAPI household questionnaire could also be completed by other adults in household (16 or over, including cohort member).

‡ Parent web survey currently in the field.

§ Maximum of two older siblings selected.

CAPI, Computer assisted personal interview; CASI, Computer assisted self-interview; CAWI, Computer assisted web interview.

### Parent and cohort member reports

The principal reporters throughout childhood and adolescence were cohort members' main residential parent (predominantly mothers), the main parent’s residential partner (typically fathers), and the cohort members themselves. Parents provided extensive information regarding family structure and socioeconomic circumstances, parenting practices and family environment, and the physical and mental health of the participant’s parents. The parents also reported on children's development, including physical and mental health, childcare and schooling, and leisure activities.

Cohort members began contributing directly to data collection at age 3 years through physical measurements and cognitive assessments. From age 7 years, cohort members also completed self-report questionnaires covering hobbies, friendships, school experiences, and mental health and wellbeing. During this sweep, participants also wore an accelerometer to capture physical activity. With increasing maturity, the content of child self-report expanded. From 11 years of age, cohort members reported on family relationships, experiences of bullying and victimisation, antisocial behaviours, and social media use. At age 14 years, the survey incorporated questions on puberty and sexual relationships, self-harm, as well as ethnicity, sexual identity, gender identity. Cohort members wore accelerometers during this sweep and, for the first and only time, completed a time use diary on how they spent their time. Participants (and their biological parents) also provided saliva samples for DNA extraction. By age 17 years, measures of cohort member’s employment and income were added to data gathered on other measures also administered at previous ages, and fewer data were gathered by parental reporting on cohort members. All data collection sweeps from ages 9 months to 17 years were done in the home. At age 23 years, sequential mixed mode was used for data collection (online, then face to face), after experimental evidence from a soft launch phase showed that this approach achieved higher response rates than face to face interviewing alone. Cohort members became the main respondents at this age, reflecting their transition into independent adulthood.

Data gathered during the sweep at age 23 years retained many existing domains, but the survey was expanded considerably to capture early adult trajectories, including education and training after the age of 16 years, employment, housing, partnerships, and fertility. Also surveyed were the participants’ cohabitating partners, which gave parallel information on the socioeconomic profiles, health profiles, and family backgrounds of the cohabiting partners.

A short online survey is currently underway involving parents and carers to provide additional insight into the data collected at participant age 23 years from people who play a parental role in cohort member’s lives. These data will become available by the end of 2026.

### Other contributors

Additional contributors enriched earlier surveys. Up to two older siblings aged 10-15 years completed questionnaires when cohort members were aged 3 and 5 years, reporting on their own activities, home life, health, and experiences of school, bullying and victimisation, and antisocial behaviours. Teachers provided assessments at participant ages 5, 7, and 11 years, offering external reports on children's behaviour, development, and educational context. Interviewers also recorded structured observations of the home and neighbourhood environments at participant ages 3 and 5 years.

### Biological samples

Biological samples were collected at several stages: at age 3 years, oral fluid swabs were taken from cohort members to assay antibodies to viral infections, and at around age 7 years, parents were invited to mail children's naturally shed milk teeth for assessment of exposure to lead. At age 14 years, saliva samples from cohort members and their biological resident parents were collected for DNA extraction. Genomic data are available through the Centre for Longitudinal Studies Data Access Committee, and polygenic scores have also been derived and made available to researchers, currently covering 44 traits (with further scores in development) spanning anthropometry (body measurements), brain structure and cognition, health behaviours, mental health, personality, physical health, and social outcomes.^7^

### Linked administrative data

Consent has been obtained to link administrative records to the survey data, with current and upcoming linkages (table 3). This approach enables an ongoing programme of linkages to health and economic records for parents and cohort members, as well as education, student loans, and criminal justice records for cohort members. Consent rates and successful linkage rates are generally very high (table 3).

**Table 3:** Administrative data linked to the MCS

Type of administrative data		Access and availability
**Linkage data**		
Education records (primary/secondary school)	Parental consent rate at participant age 7 years: 94% England (National Pupil Database, School Census 2010): KS1-KS5 (ages 5-18 years), linkage rate 89% England: foundation stage profile and teacher survey: age 5 years (year 2006) Wales: National Pupil Database, KS1-KS4 (age 5-16 years), linkage rate 100% Wales: Welsh Examinations Database (WED), education after 16 years of age Scotland: Pupil Census 2008 for year 2008-09 and attendance and absence for year 2008-09: linkage rate 92% Scotland: School Education Records, age 4-16 years	UKDS UKDS UKDS and SAIL Databank SAIL Databank UKDS UKDS (from spring 2026)
Higher education records	Cohort member consent at age 17 years: 88% consent rate Higher Education Statistics Agency (HESA)	Linkages yet to be done
Health records	Parental consent at age 7 years: 93% consent rate Cohort member consent at age 17 years: 86% consent rate England: Hospital Episode Statistics (HES) (2000-23), 81% linkage rate in 2020, 53% linkage rate in 2024 Wales: Patient Episode Database for Wales (PEDS) (2001-2012): 100% linkage rate. Scotland: Scottish Medical Records (SMR) (2000-2015), 99% linkage rate	UKDS until early 2027, UK LLC thereafter UKDS, SAIL Databank (for later data) UKDS
Birth registration	Consent at age 9 months: 90% consent rate, 89% linkage rate England and Wales: Office for National Statistics (ONS) data Scotland: General Register Office (GRS) data Northern Ireland: Northern Ireland Statistics and Research Agency (NISRA) data	UKDS UKDS UKDS
Maternity hospital records	Parental consent at age 9 months: 90% consent rate, 75% linkage rate England: Hospital Episode Statistics (HES) data Wales: Health Solutions Wales (HSW) data) Scotland: Information and Statistics Division (ISD) data Northern Ireland: Department of Health, Social Services and Public Safety (DHSSPS) data; Health and Social Services (HSS) board data	UKDS UKDS UKDS UKDS
Economic records	Cohort member consent at age 17 years: 83-84% consent rate Her Majesty's Revenue and Customs (HMRC); linkages yet to be done Department for Work and Pensions (DWP) / Northern Ireland Department for Communities, Social Security Agency (SSA); linkages to be enacted	UK LLC (from summer 2026) UK LLC (from summer 2026)
Student loans	Cohort member consent at age 17 years: 84% consent rate Student Loans Company (SLC)	UKDS (from summer 2026)
Crime records	Cohort member consent at age 17 years: 83% consent rate Police National Computer (PNC) held by Ministry of Justice; linkages yet to be done	Linked for specific research project at Office for National Statistics Secure Research Service
**Geographical data** *		
Geographical identifiers	Geographical identifiers for linking with other geographical data. Sweeps 1-7 (age 9 months to 17 years). Age 23 years forthcoming. Data: British National Grid, Irish National Grid	UKDS
Distance to school	Distances to current, first, second, and third choice school. Sweep 3 (at age 5 years). Distances between home and school. Sweeps 3-6 (ages 5-14 years). Data: Ordnance Survey National Grid coordinates	UKDS UKDS
Distance to grammer schools	Distance to English grammar schools. Sweep 5 (age 11 years) Data: Ordnance Survey Integrated Transport Network	UKDS
Home move distance	Home move distance between sweeps. Sweeps 1-6 (age 9 months to 14 years) Data: British National Grid coordinates, Irish National Grid coordinates.	UKDS
Green space availability	Green space deciles. Sweeps 1-6 (9 months to age 14 years) Data: Generalised Land Use Database (GLUD)	UKDS
Air pollution	Air pollution deciles. Sweeps 1-6 (9 months to age 14 years) Data: Multiple Environmental Deprivation Index (MEDix)	UKDS

Key stage 1 includes ages 5-7, key stage 2 includes ages 7-11 years, key stage 3 includes ages 11-14 years, key stage 4 includes age 14-16 years, and key stage 5 includes ages 16-18 years.

* This data deposit did not require participant consent, as the linkage is performed at the school level rather than the individual level.

KS, key stage; SAIL, Secure Anonymised Information Linkage (Wales); UKDS, UK Data Service.

Current linked health datasets available through UK Data Service include Hospital Episode Statistics for England, Scottish medical records, and Welsh health records, as well as birth registration and maternity hospital records for all four nations. Availability varies by country, with long term access to updated data provided through the UK Longitudinal Linkage Collaboration and, for health data in Wales, the Secure Anonymised Information Linkage databank.^8–10^

Linkage to education records for cohort members is also in place, accessible through the UK Data Service, and through the Secure Anonymised Information Linkage databank for Wales which includes also A level results (education after 16 years of age).^11^ These datasets include the National Pupil Database for England and Wales, the Scottish Pupil Census 2008, and (from 2026) linked Scottish school education records, as well as Student Loans Company records for England and Wales. Economic records have also been linked and will be made available through the UK Longitudinal Linkage Collaboration in the near future.

In addition, a range of geographical data sources have been linked to the MCS (table 3), including geographical identifiers, distance to school, distance to grammar schools, home move distance, green space availability, and exposure to air pollution.

## Missing data

Missing data are a challenge in all longitudinal studies because some participants drop out over time, shown in the MCS by declining sample sizes across sweeps (figure 1). Attrition is driven primarily by refusal: for example, by the sweep at age 5 years, 12.0% of the original sample was lost to refusal, compared with 5.7% due to participants being untraceable or uncontactable, and 1.5% due to ineligibility resulting from death or emigration.

Attrition disproportionately affects vulnerable and disadvantaged groups.^12,13^ Recent systematic identification of sweep specific non-response in the MCS shows that the most consistent predictors include lower socioeconomic status, participants’ parents not currently having paid employment, parents not participating in general elections, and cohort members who were not breastfed.^13^If not handled properly, attrition can bias analyses by making the sample less representative of the original cohort.

To counteract this potential bias, the MCS gives attrition weights with each data deposit—combined with the original survey design weights—for each sweep. These inverse probability weights are based on models predicting non-response using information from earlier sweeps. The user guides for each sweep describe how the weights were constructed: for example, the attrition weights for participants aged 23 years incorporated cohort member sex, ethnicity, country of residence at entry to study, cognitive ability and conduct and emotional problems in childhood, whether they were breastfed, mother's age at first birth, mother's mental health at participant age of 9 months, and number of parents in household, housing tenure, accommodation type, parental education and occupational status of the parent at participant age 11 years. Applying these weights results in sample characteristics of participant age 23 years (pale yellow bars) that closely match those at baseline (pale blue bars, figure 2).

Another approach to handling missing data is multiple imputation, which restores missing values using observed data to predict non-response. When the missingness is predictable from observed variables, mutiple imputation can yield efficient population estimates,^12^ and improves statistical power by retaining a larger effective sample size than inverse probability weighting. When using multiple imputation, data users should still apply survey weights in their analysis, and if imputations are not performed on the full initial sample, the attrition weight should be applied to the sweep to which imputations are made. The recent work on predictors of non-response across all MCS sweeps^13^ can inform multiple imputation models and support the development of more precise attrition weights. A comprehensive guide to handling missing data in Centre for Longitudinal Studies cohort studies—including instructions for setting up multiple imputation models and generating custom inverse probability weights—is also available.^12^

## Navigating MCS datasets

The data available in the MCS are very extensive and identifying exactly which variables and measures are available and when they were collected can pose a challenge for prospective researchers. A range of resources exists to help users navigate the MCS data, such as questionnaires, user guides, and technical reports. Several tools also support variable search. The MCS Excel data dictionary provides detailed information on over 20 000 variables (eg, topic, labels, and response categories) and a searchable tool (CLS Explore) based on this dictionary has also been developed. For cross study searches, the CLOSER Discovery platform can be used. The Catalogue of Mental Health Measures offers a dedicated search tool for mental health and wellbeing measures used across longitudinal studies based in the UK, including all such measures in the MCS. All resources are available through the Centre for Longitudinal Studies website.^14^

A new MCS core data file is available, combining all seven childhood and adolescent sweeps and containing about 170 key variables including, but not limited to, demographic data, physical and mental health, educational outcomes, body measurements, and cognitive tests. The file will be updated as new sweeps of data are released. Researchers can also create bespoke versions using the accompanying STATA and R code, which allows additional variables to be incorporated and the dataset to be regenerated in customised form.

## Data availability

The MCS is run by the Centre for Longitudinal Studies at University College London, which collects and prepares the data before depositing it with the UK Data Service for wider use in research.^15^ Most MCS survey data can be downloaded immediately after registering with the UK Data Service and agreeing to the standard end user licence. Some datasets, such as polygenic scores, require a special licence and additional application steps, after which the data can be downloaded. More sensitive data—including most linked administrative records—are available under secure access (controlled data) and require a formal application, specialist training, and access through a secure research environment. A small number of specialised datasets are hosted by other public repositories. For those looking to use MCS data linked with updated administrative data, cohort data can be obtained through the UK Longitudinal Linkage Collaboration,^16^ and for Welsh data also through the Secure Anonymised Information Linkage databank. Researchers may also apply directly to Centre for Longitudinal Studies for materials not available elsewhere, such as biological samples and genetic data. Full details and access pathways are provided on a dedicated Centre for Longitudinal Studies webpage.^17^

## Strengths and limitations

The main strength of the MCS lies in the study design: a large birth cohort study with multidisciplinary data collected from participants across their lifespan from early childhood to the most recent sweep at early adulthood, which enables examination and analyses that are not possible in many other large, cross sectional UK surveys or studies that have not followed the same cohort of participants from birth. The MCS also offers several advantages over earlier UK birth cohort studies: for example, data reported directly by fathers, an ethnically diverse participant sample, and representation from all four UK nations. The study also conducted frequent sweeps during childhood, and incorporated gold standard measures that are widely used in child development research, enabling detailed investigation of early life development. In addition to introducing novel topics not covered in previous cohort studies, the MCS repeats many core measures, facilitating cross cohort comparisons. The longitudinal study design, which repeatedly collects data on many measures over time, supports stronger causal inferences by enabling within person analyses, incorporating extensive information on confounding variables, and establishing clear temporal ordering between exposures and outcomes.

However, limitations also exist: the multidisciplinary nature of the MCS means that, although the study covers a wide range of domains, the depth of measurement within each topic is constrained by questionnaire length and the need to reduce participant burden. As with all longitudinal studies, attrition remains an ongoing challenge, underscoring the importance of carefully resolving the issue of missing data. Finally, the volume and complexity of the datasets—including the presence of twins and triplets—requires strong skills in data merging, management, and longitudinal analysis.

## Patient and public involvement

Patient and public involvement throughout the MCS has been incorporated. For instance, a participant engagement survey conducted before the age 14 sweep gathered cohort members' experiences of taking part and views on how engagement and retention could be improved over the long term the results of which directly informed the design of the survey at subsequent survey sweeps. Focus groups with people of a similar age have also been conducted before undertaking surveys to ensure the study content and approach remain relevant and appropriate. To strengthen ongoing involvement, a dedicated MCS participant panel is being established that will provide regular input from cohort members into study development.

In addition, the Child of the New Century website supports continued engagement by keeping cohort members updated on study findings, new developments, and opportunities to get involved.

## Findings from the cohort

The MCS has become one of the most widely used sources of evidence on children and families in the UK. The study data are routinely analysed by researchers across academia, central government, think tanks, and the non-profit sector. According to the Centre for Longitudinal Studies bibliography (which catalogues all publications based on cohort studies managed by the Centre for Longitudinal Studies) more than 1600 published outputs have used MCS data.^18^ Publications drawing on the MCS cover a wide range of domains between disciplines that reflect the development of children born at the turn of the millennium in the UK over their lifespan. Key themes include social determinants of health, health behaviours, physical health, mental health and wellbeing, cognitive development, educational trajectories, family processes and parenting, adolescent development, and methodological and measurement research, among others. The recent release of the data from participants aged 23 years provides important new opportunities to extend this work, particularly in relation to early adult outcomes and transitions into adulthood.

The study is highly trusted by policymakers: government departments and devolved administrations frequently engage with MCS findings and rely on MCS evidence when designing and evaluating policies.^19^ The study has influenced UK policy in numerous areas of family and child wellbeing: MCS evidence has directly informed the development of the Domestic Abuse Act by highlighting that children living in households affected by domestic abuse are at increased risk of behavioural and conduct problems during adolescence,^20^ with MCS findings playing a key role in lobbying the government to recognise children who witness domestic abuse as victims.^21^ Research from the study has also evidenced inequalities in breastfeeding support,^22^ to strengthen investment in breastfeeding support initiatives during early childhood. MCS analyses have also underpinned improvements in mental health and wellbeing support for children and young adults, providing policymakers with robust, longitudinal evidence on risk, resilience, and service needs in this group.^23,24^

Beyond academic and policy, the study has also received extensive coverage in the media, increasing public understanding of children's lives in the UK today.

## Future plans

Scientific development for the next planned sweep of the study, at participant age 27 years, is currently underway. Development plans include consulting the scientific community and other stakeholders on survey content in response to emerging priorities in lifecourse studies (eg, health, employment, relationships, and wellbeing), and exploring the collection of data on the next generation as an increasing number of cohort members are having children.

The MCS website provides comprehensive information on the study, including questionnaires and other study documentation, and recent news and updates.^25^
