# Supplementary material for: Millennium Cohort Study: cohort profile and data resource update
Source: BMJ Med. 2026 Sep 18;5(1):e002429. doi: 10.1136/bmjmed-2025-002429 (PMC13599884; doi:10.1136/bmjmed-2025-002429)
Supplement: Supplementary file 1 [file bmjmed-2025-002429.reviewer_comments.pdf]

PEER REVIEW HISTORY

BMJ Medicine publishes all reviews undertaken for accepted manuscripts. Reviewers are asked to complete a checklist review form and are provided with free text boxes to elaborate on their assessment. These free text comments are reproduced below.

ARTICLE DETAILS

**TITLE (PROVISIONAL)**	Cohort and Data Resource Profiles Cohort profile: Millennium Cohort Study
**AUTHORS**	Villadsen, Aase; Fitzsimons, Emla

VERSION 1 - REVIEW

**REVIEWER NAME**	Harron, Katie
**REVIEWER AFFILIATION**	
**REVIEWER CONFLICT OF INTEREST**	I am a co-I and co-author of one of the authors (Emla Fitzsimons). I don't think that this influences my ability to provide an impartial review.
**AI DISCLOSURE**	No
**DATE REVIEW RETURNED**	16-Dec-2025

**GENERAL COMMENTS**	I think this is an excellent data resource profile which provides a lot of useful information on a complex dataset. I have a few queries:<br><br>For an international audience, it would be helpful to explain what the Child Benefit register is (or at least, who would be included/excluded in it as a sampling frame).<br><br>The Data availability section doesn't mention UKLLC, but this is mentioned under linked administrative data. Could this be clarified?<br><br>Missing data – do users combine the use of survey / attrition weights with multiple imputation or is it recommended that only one of these approaches is used?<br><br>Linked admin data section – can you give some figures here on the proportion with linked data, and explain that this is not available for everyone given consent and linkage issues?<br><br>Figure 1 – can this have a labelled y axis?<br><br>Figure 2 – in footnote give NVQ in full

**REVIEWER NAME**	Davies, Neil
**REVIEWER AFFILIATION**	UCL, Division of Psychiatry and Department of Statistical Science
**REVIEWER CONFLICT OF INTEREST**	The authors are colleagues and in the case of Emla a co-author of mine. However, I do not work closely with Emla and do not believe that this affected my review of this profile.
**AI DISCLOSURE**	No
**DATE REVIEW RETURNED**	22-Dec-2025

**GENERAL COMMENTS**	This is a comprehensive and very well written cohort profile that should be very useful for researchers. I do not have substantive comments on the manuscript, it covered all the relevant areas. One optional addition would be a figure illustrating when each of the waves of the survey occurred relative to follow-up – a bit like Figure 1 in Shireby et al. (2025). This could be added to by adding a summary of the years for which linked data are available, for example. However, this is optional, and as the paper stands, it would make a very useful addition to the literature.<br><br>I would suggest adding the following reference to the biological samples section:<br>1<br>Shireby G, Morris TT, Wong A, et al. Data Resource Profile: Genomic data in multiple British birth cohorts (1946–2001)—linkage with health, social, and environmental data from birth to old age. International Journal of Epidemiology. 2025;54:dyaf141. doi: 10.1093/ije/dyaf141<br><br>For the LLC could use the following reference:<br>1<br>Boyd A, Evans K, Turner EL, et al. UK Longitudinal Linkage Collaboration (UK LLC): The National Trusted Research Environment for Longitudinal Research. IJPDS. 2025;10. doi: 10.23889/ijpds.v10i1.2468

**REVIEWER NAME**	Islam, Nazrul
**REVIEWER AFFILIATION**	University of Southampton, School of Primary Care, Population Sciences and Medical Education
**REVIEWER CONFLICT OF INTEREST**	None
**AI DISCLOSURE**	No
**DATE REVIEW RETURNED**	05-Jan-2026

**GENERAL COMMENTS**	Thank you for the opportunity to review the manuscript which is written well. I hope the authors will find the following comments and suggestions useful for their revision.<br><br>It is not clear if/how this profile adds new information to the previous cohort profile.<br><br>A head-to-head comparison of the demographic characteristics with the national population (at the corresponding/nearest calendar year) will should if the samples are representative/ generalisable or if additional extrapolation is needed.<br><br>Some additional benchmarking with the national estimates could add more values and confidence. For example, the incidence and/or prevalence of certain condition(s), e.g., substance use, mental health, estimated from this cohort and how those compare against the national estimates from other sources (such as census).<br><br>Could some info be included on rural/urban distribution? Does it data on IMD?<br><br>It says, "...the design oversampled children in socioeconomically disadvantaged areas." Could the authors clarify how disadvantaged areas were defines, for example, was it based on IMD?

	Could the data be linked with other resources, let's say, hospital admission (HES) data, primary care data, more importantly, mortality data (ONS)?<br><br>Minor: could we use alternative words such as waves rather than 'sweeps'?

**REVIEWER NAME**	Emmerson, Chris
**REVIEWER AFFILIATION**	Public Health Wales, Health Improvement Division
**REVIEWER CONFLICT OF INTEREST**	None
**AI DISCLOSURE**	No
**DATE REVIEW RETURNED**	30-Jan-2026

**GENERAL COMMENTS**	Overview<br>This paper provides a description of the cohort included within the Millennium Cohort Study (MCS). The paper notes two previous papers which described the MCS:<br>• Joshi and Fitzsimons (2016) The UK Millennium Cohort Study: the making of a multipurpose resource for social science and policy in the UK<br>• Connelly and Platt (2014) Cohort Profile, UK Millennium Cohort Study<br>A brief review of studies using the MCS and published in the BMJ up to 2025 all used one of these papers. There have been multiple sweeps since publication of those papers, and the cohort have aged out of secondary education and into adulthood.<br>UCL, which conducts the MCS also has resources publicly available via the Centre for Longitudinal Studies site here: https://cls.ucl.ac.uk/cls-studies/millennium-cohort-study/ (Reference 7 in this paper). However, this website does not have a succinct and peer-reviewed summary of the study.<br>There therefore is a clear rationale for a paper describing the MCS to be published in the BMJ.<br>In general, the paper describes the MCS clearly and in an appropriate level of detail for BMJ readers with an interest in longitudinal research, or who are reviewing research using the MCS to gain insights into what is included in the data and how it was gathered. However, the paper would benefit from some restructuring and additional material on some key points.<br>Major comments<br>Purpose and target audience<br>It is assumed that the paper is aimed at public health researchers who are either considering using MCS data for longitudinal research or seeking to understand the basic methods and value of the MCS in order to engage with research that draws on this resource. It would be helpful to state clearly the audience for the paper.<br>Structure<br>There is some inconsistency in header and subheader formatting and these could be amended to better organise the material and guide the reader. The first header is ‘The Millennium Cohort Study’: this would be better titled ‘Introduction’ or similar. ‘Population’ and ‘Measures’ appear to be headers, with subsequent section headings italicised as though they are subheaders of measures, which may be the case to ‘Biological samples’ but not beyond. ‘Missing data’ would be better presented after ‘Measures’. ‘Strengths and limitations’ and ‘Findings from the cohort’ would be better situated

	earlier in the paper, above sections which cover administrative and ethical dimensions such as data availability and patient and patient and public involvement.<br>The ‘update’ section would be better integrated in introductory paragraphs; in addition, the first reference (Ref 2, Fitzsimons et al 2025) does not appear to be a description of the cohort which this paper is updating.<br>Population<br>In this section the authors note describe the sampling procedure as “universal Child Benefit register served as the sampling frame. Residential areas (electoral wards) were selected, and all eligible children within them were recruited.” As written, this suggests that all children born in the sampling period and included on the Child Benefit register were included in the sample, which is not accurate. As stated on the CLS MCS site “The sampling strategy was to make a selection of areas of residence, and within them to recruit 100 per cent of the children born in the eligible period. The statistical geography available for such clusters in 2000-01 was the boundaries of electoral wards as they stood before updating at the 2001 Census.” The text in this paper should reflect this more detailed description of the sampling strategy and also describe how areas of residence were selected for sampling (e.g. was selection randomised, stratified, etc).<br><br>There is a small discrepancy between the final sample size reported in this paper (19,244 families, 19,519 children) and that reported on the CLS MCS site (19,243 families, 19,517 children). This should be resolved or addressed.<br><br>As the samples selected for to explore the impact of the COVID-19 pandemic were smaller than the main sample, the method of selecting these smaller cohorts should be described.<br><br>Child benefit was universal at the point of recruitment and it is assumed that almost all families will have claimed this benefit. However, it would be helpful to clarify (1) that this was a universal benefit at the time (given that this has not been the case since 2013 and the wording does not clearly link universality to the benefit, rather than just the register) (2) provide a measure or estimate of child benefit uptake and therefore inclusion on the register in the recruitment period, and noting any evidence of possible selection biases.<br><br>The rationale for oversampling certain categories children (specified as those living in disadvantaged areas, UK nations other than England and from ethnic minority backgrounds) notes a desire to increase analytic and concerns that these children are typically under-represented groups. This suggests two distinct rationales, which may apply differently for different groups: sample sizes may be smaller, so larger samples are required to provide meaningfully precise estimates (‘increase analytic power’) and a concern that those from some backgrounds may be more likely to drop out (‘typically underrepresented groups’). These rationales may have different relevance to different groups (there are clearly more children in England than in Scotland/Wales/NI, but they are not inherently more likely to drop out; area based measures of deprivation often divide the population into quantiles, so they are evenly spread, but attrition may be higher). Greater clarity here would benefit the reader.

	"Residential areas (electoral wards) were selected…." 'Residential areas' seems redundant (at the point of recruitment, ONS geographies were based on electoral wards, which is a clearly defined term and includes the entire population, as every individual lives in an electoral ward. Residential areas could be interpreted as areas with a high proportion of residential properties (e.g. suburbs but not city centres), so could usefully be removed as a term.<br><br>Linked administrative data<br>"Current linked health datasets include Hospital Episode Statistics (HES) for England, Scottish medical records, and Welsh health records." It would be helpful to clarify the specific datasets and/or data that is included, in particular in UK nations other than England. For example, for Wales the equivalent of HES is the Patient Episode Database for Wales (PEDW), available via the SAIL databank, as described in Kerry-Barnard et al (2024), Reference 3 in this paper. A link to a resource that provides details of specific datasets included alongside the MCS would also be useful (e.g. the Health Data Research Gateway, https://healthdatagateway.org/en)<br><br>Parent and cohort member reports<br>This section provides a succinct and well structured description of the measures collected, at what point they were collected and from whom and how data were gathered.<br><br>Data availability<br>This section would benefit from more structure that relates (1) categories of measures (2) access conditions (it might be helpful to relate this to the UK Data Services descriptions of access restrictions) (3) data holders (e.g. linked data for Welsh children will be accessed through the SAIL databank).<br>Strengths and limitations<br>It would be particularly helpful for researchers considering whether the MCS would meet their needs to include some brief comparisons with other longitudinal datasets such as ONS cross-sectional surveys and the UK Household Longitudinal Study (UKHLS). The availability of family and educational data, and the length of the study at this point in time are all particular strengths of the study and it is useful to see these stressed.<br><br>Findings from the cohort<br>The paper usefully provides examples of studies that have used the MCS across a range of topics. As BMJ readers are likely to have a wide range of research interests and researchers are likely to be looking for application to specific questions, this approach might be even more effective if studies were organised by theme including examples from both topic areas (e.g. health behaviours, social determinants of health) and research designs (e.g. multilevel models, case control studies). The fact that the data describe a cohort that is now entering a life stage that where they will be engaging for the first time in activities (such as entering full time employment and starting a family) that will shape many of their future health outcomes makes the MCS of particular interest to researchers across a broad range of areas.<br><br>Missing data<br>This section should provide more discussion of the data presented in Figure 1, in particular the biases that differential rates of attrition for different groups might introduce. There is commentary on the increased likelihood of attrition amongst those from 'vulnerable and

	disadvantaged groups' but there could be some discussion of which groups this might affect particularly and why – e.g. is attrition more likely amongst families of lower socioeconomic status, ethnic minority backgrounds or single parent families? Is this due to refusal to take part in subsequent sweeps, moving home or other reasons?<br><br>References<br>Reference 20 - Narayanan M, Villadsen A, Katsoulis M, Dodgeon B, Ploubidis GB, Silverwood RJ, Fitzsimons E. A data driven approach to handling missing data in the UK Millennium Cohort Study. 2025 does not include journal / report details and could not be located<br><br>Minor comments<br>Population<br>Strengths and limitations of the cohort data<br>It would be helpful to clarify the term 'gold standard' as used here; does the term simply refer to measures generally used and with accepted and consistent definitions?<br><br>References<br>References would be improved by adding DOIs and/or urls where available.

VERSION 1 – AUTHOR RESPONSE

Reviewer: 1
Prof. Katie Harron
Comments to the Author
I think this is an excellent data resource profile which provides a lot of useful information on a complex dataset. I have a few queries:

For an international audience, it would be helpful to explain what the Child Benefit register is (or at least, who would be included/excluded in it as a sampling frame).

That is a really good point and we have now explained this in the paper. p.3: 'The sampling frame was the universal Child Benefit register, where anyone in the UK with a child can register to receive non-means tested monthly payment to help with the cost of bringing up a child'

The Data availability section doesn't mention UKLLC, but this is mentioned under linked administrative data. Could this be clarified?

Another good point. We have now included this in the data availability section, where we have mentioned both the UK LLC and also SAIL Databank.

Missing data – do users combine the use of survey / attrition weights with multiple imputation or is it recommended that only one of these approaches is used?

This is a really important point to clarity, which we have now incorporated in the paper: p8: 'When using the MI approach, data users should still apply survey weights in their analysis, and in instances where imputations are not performed on the full initial sample, also attrition weight for the sweep to which imputations are made should be applied'

Linked admin data section – can you give some figures here on the proportion with linked data, and explain that this is not available for everyone given consent and linkage issues?

This is a good point. Our presentation of the linked data was previously part of Table 1. We have now made a dedicated table for linked data (Table 3) where we have provided more information, including consent rates and linkage rates (where available).

Figure 1 – can this have a labelled y axis?

A label has now been applied: 'Sample size of cohort members'

Figure 2 – in footnote give NVQ in full

A foot note has now been applied. 'NVQ=National Vocational Qualifications. NVQ1 (CGSEs graded less than C); NVQ2 (CGSEs graded C or above); NVQ3 (A or AS level); NVQ4 (Degree); NVQ5 (Postgraduate).'

Reviewer: 2
Prof. Neil Davies, UCL
Comments to the Author
bmjmed-2025-002429: Cohort and Data Resource Profiles Cohort profile: Millennium Cohort Study
This is a comprehensive and very well written cohort profile that should be very useful for researchers. I do not have substantive comments on the manuscript, it covered all the relevant areas. One optional addition would be a figure illustrating when each of the waves of the survey occurred relative to follow-up – a bit like Figure 1 in Shireby et al. (2025). This could be added to by adding a summary of the years for which linked data are available, for example. However, this is optional, and as the paper stands, it would make a very useful addition to the literature.

We think perhaps a new figures will be repeating some of the information in Figure 1. It will also be very tricky and messy to add the years for linked data, as this varies for each type of linked data, and also by country. However, we have now made a dedicated table just for the linked data (Table 3), where we have provided a lot more detail about what is covered, by country where linkages often vary, and providing details on time period/sweep/age.

I would suggest adding the following reference to the biological samples section:
1
Shireby G, Morris TT, Wong A, et al. Data Resource Profile: Genomic data in multiple British birth cohorts (1946–2001)—linkage with health, social, and environmental data from birth to old age. International Journal of Epidemiology. 2025;54:dyaf141. doi: 10.1093/ije/dyaf141

For the LLC could use the following reference:
1
Boyd A, Evans K, Turner EL, et al. UK Longitudinal Linkage Collaboration (UK LLC): The National Trusted Research Environment for Longitudinal Research. IJPDS. 2025;10. doi: 10.23889/ijpds.v10i1.2468

Great many thanks. Both references have now been added, many thanks for these suggestions.

Reviewer: 3
Dr. Nazrul Islam, University of Southampton

Comments to the Author
Thank you for the opportunity to review the manuscript which is written well. I hope the authors will find the following comments and suggestions useful for their revision.

It is not clear if/how this profile adds new information to the previous cohort profile.

This information was in the section called 'Update': 'This cohort profile updates the three previous profiles, which described the MCS up to ages 11, 14, and 17, respectively (1-3). It provides the first comprehensive account of the cohort and all data resources through to the most recent sweep at age 23.'

However, we have now removed this very short section and instead provided this information in a new introductory section which provides an outline of the cohort profile.

A head-to-head comparison of the demographic characteristics with the national population (at the corresponding/nearest calendar year) will should if the samples are representative/ generalisable or if additional extrapolation is needed.

This is a good suggestion. We have now used data from the 2001 Census to compare the MCS to baseline child ethnicity and parental educational qualifications.

Some additional benchmarking with the national estimates could add more values and confidence. For example, the incidence and/or prevalence of certain condition(s), e.g., substance use, mental health, estimated from this cohort and how those compare against the national estimates from other sources (such as census).

This is a good idea. We have now compared overweight/obesity at school reception age as there is data available with a large sample size (N=435,927), although from England only, but we have run our comparative MCS analyses just for England.

Could some info be included on rural/urban distribution? Does it data on IMD?

Geographical data is not collected in the MCS surveys as such but measures such as IMD and rural/urban distinctions have been obtained using linked data, currently only available in the MCS datasets from sweep 1 (age 9 months) to 5 (age 11). We therefore cannot include this in the Figure 2 as this shows characteristics both at baseline and at age 23.

However, the reviewers feedback has us made aware that we in our cohort have not covered the geographical data available liked to the MCS. These are now included in Table 3, which is a new table dedicated just to linked data, which before were shown in Table 1.

It says, "...the design oversampled children in socioeconomically disadvantaged areas." Could the authors clarify how disadvantaged areas were defines, for example, was it based on IMD?

We have now added that definition of disadvantaged areas is based on the Child Poverty Index. For completion we have also clarified that oversampling of ethnic minorities were done by oversampling areas with a high ethnic minority population using the 1991 Population Census.

P3: 'the design oversampled children in socioeconomically disadvantaged areas (using the Child Poverty Index), children from areas with a high ethnic minority population (in England) using the 1991 Population Census, and children living in Scotland, Wales, and Northern Ireland.'

Could the data be linked with other resources, let's say, hospital admission (HES) data, primary care data, more importantly, mortality data (ONS)?

The MCS has already been linked with HES. Table 3 (new table) now provides a lot more detail on the linked data.

Minor: could we use alternative words such as waves rather than 'sweeps'? [TN - editorial note: we can be guided by the term you've used in your study so far]

The MCS (study documentation, previous cohort profiles, website) tends to use the word sweep rather than wave. Therefore, we would like to retain this word.

Reviewer: 4
Mr. Chris Emmerson, Public Health Wales
Comments to the Author
Cohort profile: Millennium Cohort Study
Review – 25 Jan 2026

Overview
This paper provides a description of the cohort included within the Millennium Cohort Study (MCS). The paper notes two previous papers which described the MCS:
• Joshi and Fitzsimons (2016) The UK Millennium Cohort Study: the making of a multipurpose resource for social science and policy in the UK
• Connelly and Platt (2014) Cohort Profile, UK Millennium Cohort Study
A brief review of studies using the MCS and published in the BMJ up to 2025 all used one of these papers. There have been multiple sweeps since publication of those papers, and the cohort have aged out of secondary education and into adulthood.
UCL, which conducts the MCS also has resources publicly available via the Centre for Longitudinal Studies site here: https://cls.ucl.ac.uk/cls-studies/millennium-cohort-study/ (Reference 7 in this paper). However, this website does not have a succinct and peer-reviewed summary of the study.
There therefore is a clear rationale for a paper describing the MCS to be published in the BMJ.
In general, the paper describes the MCS clearly and in an appropriate level of detail for BMJ readers with an interest in longitudinal research, or who are reviewing research using the MCS to gain insights into what is included in the data and how it was gathered. However, the paper would benefit from some restructuring and additional material on some key points.

Major comments
Purpose and target audience
It is assumed that the paper is aimed at public health researchers who are either considering using MCS data for longitudinal research or seeking to understand the basic methods and value of the MCS in order to engage with research that draws on this resource. It would be helpful to state clearly the audience for the paper.

This is very useful. We have now included this in the abstract.

Structure
There is some inconsistency in header and subheader formatting and these could be amended to better organise the material and guide the reader. The first header is 'The Millennium Cohort Study': this would be better titled 'Introduction' or similar. 'Population' and 'Measures' appear to be headers, with subsequent section headings italicised as though they are subheaders of measures, which may

be the case to 'Biological samples' but not beyond. 'Missing data' would be better presented after 'Measures'. 'Strengths and limitations' and 'Findings from the cohort' would be better situated earlier in the paper, above sections which cover administrative and ethical dimensions such as data availability and patient and patient and public involvement.
The 'update' section would be better integrated in introductory paragraphs; in addition, the first reference (Ref 2, Fitzsimons et al 2025) does not appear to be a description of the cohort which this paper is updating.

These suggestions make a lot on sense. We had perhaps followed too closely the subheading template and order we were provided for writing the profile. We have now done an introductory section where the update information has been included, and we have rearranged the sections in a more logical order.

The Fitzsimons et al 2025 reference describes the MCS up until previous sweep at age 17, but does not include the recent age 23 survey which is in the current cohort profile
(Fitzsimons E, Pople L, Libuy N, Calderwood L, Haselden L. Reflections on the Design and Development of First 18 Years of the Millennium Cohort Study. European Psychologist. 2025.)

Population
In this section the authors note describe the sampling procedure as "universal Child Benefit register served as the sampling frame. Residential areas (electoral wards) were selected, and all eligible children within them were recruited." As written, this suggests that all children born in the sampling period and included on the Child Benefit register were included in the sample, which is not accurate. As stated on the CLS MCS site "The sampling strategy was to make a selection of areas of residence, and within them to recruit 100 per cent of the children born in the eligible period. The statistical geography available for such clusters in 2000-01 was the boundaries of electoral wards as they stood before updating at the 2001 Census." The text in this paper should reflect this more detailed description of the sampling strategy and also describe how areas of residence were selected for sampling (e.g. was selection randomised, stratified, etc).

We have now made it clear that eligible children in the selected wards were *targeted* for recruitment. Which is of course not the same as them all actually being recruited. P3: 'Geographical areas (electoral wards as they stood before updating at the 2001 Census) were selected, and all eligible children within them were targeted for recruitment.'

We have also added some more details about the sampling approach. p.3: 'The sample of wards was selected using probability (or random) methods of selection combined with stratification and clustering. Division of the estimated achieved sample across strata was half in advantaged wards and half in disadvantaged wards in Wales, Scotland and Northern Ireland, and half in advantaged wards and a quarter each in the ethnic and disadvantaged wards in England. A detailed technical report on the sampling strategy is available (4)'.

There is a small discrepancy between the final sample size reported in this paper (19,244 families, 19,519 children) and that reported on the CLS MCS site (19,243 families, 19,517 children). This should be resolved or addressed.

Thank you for highlighting this. The real figures that on the website. Older documentation reports the slightly larger sample, but later one family was found to be a duplicate. This is now corrected across the manuscript.

As the samples selected for to explore the impact of the COVID-19 pandemic were smaller than the main sample, the method of selecting these smaller cohorts should be described.

We have now added an explanatory note to Figure 1 that explains why the sample sizes are so small in the Covid sweeps: 'The small sample sizes in the Covid sweeps were due to are due to the challenges of rapid, remote data (web survey) collection during a pandemic.'

Child benefit was universal at the point of recruitment and it is assumed that almost all families will have claimed this benefit. However, it would be helpful to clarify (1) that this was a universal benefit at the time (given that this has not been the case since 2013 and the wording does not clearly link universality to the benefit, rather than just the register) (2) provide a measure or estimate of child benefit uptake and therefore inclusion on the register in the recruitment period, and noting any evidence of possible selection biases.

Thank you for this suggestion. We have now provided a figure for take-up (97%). Very little has been published specifically in relation to take up bias of child benefits prior to the 2013 reform. However, we were able to identify one paper that had looked at take up by income quartiles showing very little variation in take up (range 95-97%). We have included this reference in the text.

The rationale for oversampling certain categories children (specified as those living in disadvantaged areas, UK nations other than England and from ethnic minority backgrounds) notes a desire to increase analytic and concerns that these children are typically under-represented groups. This suggests two distinct rationales, which may apply differently for different groups: sample sizes may be smaller, so larger samples are required to provide meaningfully precise estimates ('increase analytic power') and a concern that those from some backgrounds may be more likely to drop out ('typically underrepresented groups'). These rationales may have different relevance to different groups (there are clearly more children in England than in Scotland/Wales/NI, but they are not inherently more likely to drop out; area based measures of deprivation often divide the population into quantiles, so they are evenly spread, but attrition may be higher). Greater clarity here would benefit the reader.

This is true, in addition some groups are not just more likely to drop out but also less likely to respond to the initial survey. We had a added a sentence on p.3 that clarifies this. 'To increase analytical power for typically underrepresented groups, some of which are known to be less likely to respond initially and more like to attrit over time, the design oversampled children ….'

"Residential areas (electoral wards) were selected…." 'Residential areas' seems redundant (at the point of recruitment, ONS geographies were based on electoral wards, which is a clearly defined term and includes the entire population, as every individual lives in an electoral ward. Residential areas could be interpreted as areas with a high proportion of residential properties (e.g. suburbs but not city centres), so could usefully be removed as a term.

We have changed residential areas to geographical areas. The text now reads. p.3: 'Geographical areas (electoral wards as they stood before updating at the 2001 Census) were selected, …'

Linked administrative data
"Current linked health datasets include Hospital Episode Statistics (HES) for England, Scottish medical records, and Welsh health records." It would be helpful to clarify the specific datasets and/or data that is included, in particular in UK nations other than England. For example, for Wales the equivalent of HES is the Patient Episode Database for Wales (PEDW), available via the SAIL databank, as described in Kerry-Barnard et al (2024), Reference 3 in this paper. A link to a resource that provides details of specific datasets included alongside the MCS would also be useful (e.g. the Health Data Research Gateway, https://healthdatagateway.org/en)

Good suggestion. We have now provided the data set names. Note that there is a new table (Table 3) which is dedicated to linked data where a lot more information is now provided.

Parent and cohort member reports
This section provides a succinct and well structured description of the measures collected, at what point they were collected and from whom and how data were gathered.

Data availability
This section would benefit from more structure that relates (1) categories of measures (2) access conditions (it might be helpful to relate this to the UK Data Services descriptions of access restrictions) (3) data holders (e.g. linked data for Welsh children will be accessed through the SAIL databank).

In the data access section we just mention some types of data that fall under difference licence types, but the aim in not to list all measures in terms of how to access as this would be too cumbersome given the large number of measures and datasets. We have now fleshed out a bit more the different types of UKDS licence types (End User Licence, Special Licence, Secure Access). We have also included some more information about the data holders, including SAIL Databank.

Strengths and limitations
It would be particularly helpful for researchers considering whether the MCS would meet their needs to include some brief comparisons with other longitudinal datasets such as ONS cross-sectional surveys and the UK Household Longitudinal Study (UKHLS). The availability of family and educational data, and the length of the study at this point in time are all particular strengths of the study and it is useful to see these stressed.

Thank you for this suggestion. We have added some more here but have taken the polite approach by not directly naming other studies and data sources but just highlighting more generally the advantage that the MCS presents.

P.9: 'A main strength of the MCS is that it is a large birth cohort study with multidisciplinary data collected from the same individuals across their life course from early childhood to currently early adulthood, which enables examination and analyses that are not possible in many other large UK surveys that are cross-sectional or that have not followed the same individuals right from birth.'

Findings from the cohort
The paper usefully provides examples of studies that have used the MCS across a range of topics. As BMJ readers are likely to have a wide range of research interests and researchers are likely to be looking for application to specific questions, this approach might be even more effective if studies were organised by theme including examples from both topic areas (e.g. health behaviours, social determinants of health) and research designs (e.g. multilevel models, case control studies). The fact that the data describe a cohort that is now entering a life stage that where they will be engaging for the first time in activities (such as entering full time employment and starting a family) that will shape many of their future health outcomes makes the MCS of particular interest to researchers across a broad range of areas.

We have now added text that summarises the main themes that have been examined using the MCS. p. 10: 'Publications drawing on the MCS cover a wide and interdisciplinary range of domains that reflect the life-course development of children born in the UK at the turn of the millennium. Key themes include social determinants of health, health behaviours, physical health, mental health and wellbeing, cognitive development and educational trajectories, family processes and parenting, adolescent development, and methodological and measurement research, among others. The recent release of the age 23 data provides important new opportunities to extend this work, particularly in relation to early adult outcomes and transitions into adulthood.'

Missing data

This section should provide more discussion of the data presented in Figure 1, in particular the biases that differential rates of attrition for different groups might introduce. There is commentary on the increased likelihood of attrition amongst those from ‘vulnerable and disadvantaged groups’ but there could be some discussion of which groups this might affect particularly and why – e.g. is attrition more likely amongst families of lower socioeconomic status, ethnic minority backgrounds or single parent families? Is this due to refusal to take part in subsequent sweeps, moving home or other reasons?

We have now provided some more information about reasons for attrition using age 5 as the example. P. 7: ‘Attrition is driven primarily by refusal. For example, by the age 5 sweep, 12.0% of the sample loss was attributable to refusal, compared with 5.7% due to participants being untraceable or uncontactable, and 1.5% due to ineligibility resulting from death or emigration.’

We have also pointed to results of recent work in the MCS on non-response. P.7: ‘Recent systematic identification of sweep-specific non-response in the MCS shows that the most consistent predictors include lower socioeconomic status, parents not currently in paid employment, parental non-participation in general elections, and cohort members who were not breastfed’

References
Reference 20 - Narayanan M, Villadsen A, Katsoulis M, Dodgeon B, Ploubidis GB, Silverwood RJ, Fitzsimons E. A data driven approach to handling missing data in the UK Millennium Cohort Study. 2025 does not include journal / report details and could not be located

The paper was on the verge of preprinted when submitting the manuscript. This is now fully available online and our reference has been updated: https://osf.io/preprints/socarxiv/agqmu_v1

Minor comments
Population
Strengths and limitations of the cohort data
It would be helpful to clarify the term ‘gold standard’ as used here; does the term simply refer to measures generally used and with accepted and consistent definitions?

We have now clarified why we mean by this, adding that these are measured widely used in research on child development.

References
References would be improved by adding DOIs and/or urls where available.

We are asked to use the Vancouver style references which do not feature DOI or URLs (expect for references to websites).
